# A solid-supported membrane electrophysiology assay for efficient characterization of ion-coupled transport

**DOI:** 10.1016/j.jbc.2021.101220

**Published:** 2021-09-23

**Authors:** Nathan E. Thomas, Wei Feng, Katherine A. Henzler-Wildman

**Affiliations:** 1 Department of Biochemistry, University of Wisconsin-Madison, Madison, Wisconsin, USA; 2 Department of Molecular and Cellular Physiology, Stanford University School of Medicine, Stanford, California, USA

**Keywords:** ion-coupled transport, secondary active transport, transport assay, electrophysiology, membrane proteins, LPR, lipid-to-protein ratio, SSME, solid-supported membrane electrophysiology

## Abstract

Transport stoichiometry determination can provide great insight into the mechanism and function of ion-coupled transporters. Traditional reversal potential assays are a reliable, general method for determining the transport stoichiometry of ion-coupled transporters, but the time and material costs of this technique hinder investigations of transporter behavior under multiple experimental conditions. Solid-supported membrane electrophysiology (SSME) allows multiple recordings of liposomal or membrane samples adsorbed onto a sensor and is sensitive enough to detect transport currents from moderate-flux transporters that are inaccessible to traditional electrophysiology techniques. Here, we use SSME to develop a new method for measuring transport stoichiometry with greatly improved throughput. Using this technique, we were able to verify the recent report of a fixed 2:1 stoichiometry for the proton:guanidinium antiporter Gdx, reproduce the 1H^+^:2Cl^−^ antiport stoichiometry of CLC-ec1, and confirm loose proton:nitrate coupling for CLC-ec1. Furthermore, we were able to demonstrate quantitative exchange of internal contents of liposomes adsorbed onto SSME sensors to allow multiple experimental conditions to be tested on a single sample. Our SSME method provides a fast, easy, general method for measuring transport stoichiometry, which will facilitate future mechanistic and functional studies of ion-coupled transporters.

Ion-coupled (*i.e.*, secondary active) transporters utilize energy stored in electrochemical gradients to drive uphill substrate transport across cellular membranes. These transporters are essential to numerous physiological processes, from nutrient uptake to neural signaling, and are common drug targets, but successful therapeutic design is limited by our understanding of these integral membrane proteins’ structures, functions, and mechanisms (1, 2). Transport stoichiometry—the number of ions and substrates moved per transport cycle—is a function of the transporter’s mechanism and is a crucial determinant of the direction of driven transport, the energy spent per substrate transported, and the maximum substrate gradient that can be maintained at equilibrium. Generally, ion-coupled transporters have been assumed to operate according to tightly coupled mechanisms with a single, set transport stoichiometry. This understanding has led to the traditional classification of transporters as antiporters, symporters, or uniporters. However, there is growing evidence that many ion-coupled transporters operate through complex mechanisms that violate the assumption of stoichiometric transport, engaging in behavior that cannot be cleanly classified as antiport, symport, or uniport (3, 4, 5, 6, 7, 8). In light of these findings, there is a pressing need for detailed mechanistic investigations of a greater variety of transporters.

The emergence of high-resolution structures of membrane proteins has revealed specific substrate-binding sites on transporters, which together with biochemical assays have significantly advanced our understanding of transporter:substrate-binding stoichiometry (9, 10, 11, 12). However, it is important to distinguish binding stoichiometry from transport stoichiometry (13). At times, ions or substrates can bind as allosteric effectors without being transported (14). This is especially difficult to untangle in the case of proton-coupled transporters with multiple protonatable side chains (15, 16). Furthermore, establishing that substrate bound at a specific site can be transported is insufficient to establish that its transport is obligatory to the mechanism, as loosely coupled transporters may be able to transport one substrate without cotransport of the coupled ion or substrate (3, 17). Thus, measuring a transporter’s coupling stoichiometry not only sheds light on its biological functions, but also provides crucial information about the transport mechanism.

The only way to reliably determine transport stoichiometry is through transport assays. Reversal potential assays have emerged as the method of choice for electrogenic transporters, as they are applicable to a variety of systems and allow for model-independent determination of the transport stoichiometry (18, 19, 20, 21, 22). In brief, these assays follow transport as a function of membrane voltage for a set initial substrate and/or ion gradient. The reversal potential, the membrane voltage where there is no net transport, can be used to determine the transport stoichiometry. In theory, this method can be applied to any electrogenic transporter, but there are several important limitations. First, transport must be able to be monitored. Several eukaryotic transporters are amenable to patch clamp electrophysiology (23, 24, 25), but this technique is generally only suitable for high-flux transporters that can be highly expressed in eukaryotic cells, which excludes many structurally characterized prokaryotic transporters. Assays of purified protein in proteoliposomes allow for characterization of the widest range of transporters but require an appropriate readout of transport activity. Ideally, liposomal transport is observed through real-time probes such as fluorophores. However, fluorophores are not available for every coupling ion. Radioactive transport assays may be used instead (22) but do not offer real-time monitoring, significantly increasing the time and effort required to collect data for the multiple time points and conditions needed to determine transport stoichiometry. A second issue with traditional liposomal assays is that intraliposomal contents cannot easily be changed after reconstitution. This increases the difficulty of screening a wide range of assay conditions and can make it difficult to accurately establish internal ion and substrate concentrations. A third issue is that liposomal assays often require a large amount of purified transporters, depending on the sensitivity of the detection method and the number of conditions that must be tested. Finally, for many of the reasons listed above, traditional liposomal transport assays are low-throughput and require significant time and effort, even if the material costs can be kept to a minimum. As a consequence, transport stoichiometry has been measured for only a small fraction of structurally characterized transporters (22, 26). A method for routine measurements of transport stoichiometry would greatly facilitate functional and mechanistic studies of ion-coupled transporters.

Here we present a new approach for measuring coupling stoichiometry by adapting reversal-potential assays to solid-supported membrane-based electrophysiology (SSME), a technique developed over the last 2 decades to allow electrophysiological characterization of lower flux transporters (27, 28). SSME carries several advantages over traditional liposomal transport assays. By directly measuring transported charge, SSME allows real-time monitoring of electrogenic transport without the need for fluorophores or radiolabeled substrates. In addition, its sensitivity requires only picomole amounts of protein to achieve sufficient signal. Finally, dozens of conditions can be tested on a single sensor without the need for separate reconstitutions, greatly increasing the throughput of the assay. We demonstrate the utility of this method using *E. coli* Gdx, whose 2:1 proton:guanidinium antiport stoichiometry was recently established using traditional reversal potential measurements (29), and CLC-ec1, which has been shown to engage in both tightly coupled and loosely coupled proton:anion transport (21). With our SSME assay, we confirmed these results, using less than 2 nmol total protein to perform 400 transport assays in under a week of measurement time. In addition, we demonstrate that it is possible to change the internal ion and substrate concentrations using the SSME setup, expanding the number of experimental conditions that can be tested on a single protein sample. This assay is fast, easy, and accurate, requires a minimal amount of sample, and is broadly applicable to electrogenic transporters, regardless of coupling ion or transported substrate.

## Results

### An SSME transport reversal assay

Reversal-potential assays rely on the thermodynamic reversibility of coupled transport. That is, transport of an ion down a large electrochemical gradient can drive transport of another substrate uphill against its electrochemical gradient, and conversely, transport of a substrate down a large gradient can drive transport of an ion up its electrochemical gradient. In this respect, the distinction between “ion” and “substrate” (which can be another ion) is semantic, though in a cellular context, the driving force is most commonly provided by the proton and/or sodium electrochemical gradients. When the electrochemical gradients are balanced, no net transport occurs, allowing for calculation of the transport stoichiometry (see Experimental procedures for equations and derivations). Our assay adapts this principle to SSME.

In an SSME experiment, proteoliposomes are adsorbed onto a membrane-coated gold electrode, creating the sensor. To initiate an experiment, buffer is run over the sensor in three stages (27). First, a “nonactivating” buffer containing the same solution as inside the liposomes is flowed over the sensor to ameliorate artifacts due to buffer perfusion. Second, an “activating” buffer is flowed over the sensor to initiate transport. Capacitive coupling between the liposomal membrane and the surface-supported membrane on the electrode allows for measurement of currents across the membrane (“on-currents”). Transport proceeds until a steady state is obtained where sufficient membrane potential opposes further net transport. Third, the nonactivating buffer is reapplied, and the reverse transport process returns the sensor to its initial state. During this phase, “off-current” transport proceeds in the opposite direction of the on-current, driven by both chemical and electrical gradients.

**Figure 1:**
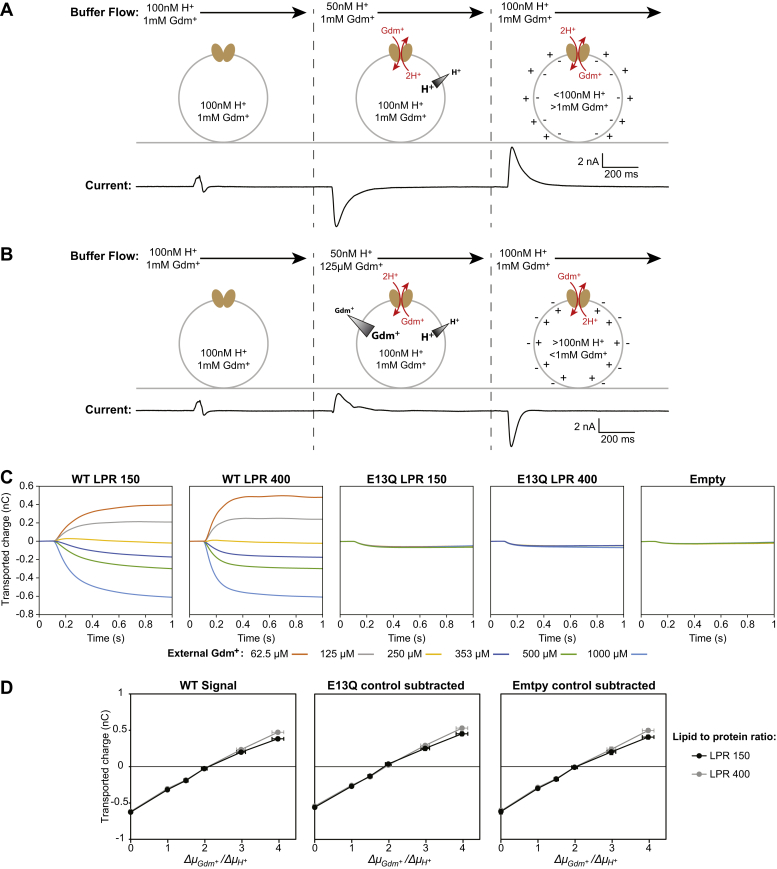
**SSME reversal assay with Gdx.** Each assay consists of three stages of buffer perfusion. In the first stage, the “nonactivating” buffer is identical to the internal buffer of the liposomes. In the second stage, the “activating” buffer sets a proton gradient and the guanidinium gradient is varied, leading to the transport on-current. In the third stage, “nonactivating” buffer is reintroduced, and the transport off-current is observed as the liposomes return to their initial state. *A*, the activating buffer sets a twofold proton gradient but no guanidinium gradient. The proton gradient drives guanidinium transport into the liposomes in exchange for two protons. Net charge is transported out of the liposomes, creating a negative on-current. *B*, the activating buffer sets a twofold proton gradient and an eightfold guanidinium gradient. The guanidinium gradient drives uphill proton transport into the liposomes, creating a positive on-current. *C*, for stoichiometry analysis, the on-current is integrated to observe transported charge. *D*, plotting integrated on-current as a function of imposed gradient ratios yields null transport at the published 2H^+^/Gdm^+^ stoichiometry, regardless of subtraction of background signal. Data points represent average normalized values obtained from four sensors for each (proteo)liposome sample. Y-error bars represent standard error of the mean, propagated where necessary, and X-error bars are calculated by propagation assuming a 2% error in substrate and ion concentrations. Complete buffer conditions can be found in Table 1.

Figure 1 shows representative traces from our assay for all three stages of the experiment. First, nonactivating buffer equilibrates the liposomes with a known concentration of proton and guanidinium. In the second stage, the activating buffer sets a twofold proton gradient to initiate transport. In Figure 1*A*, the guanidinium concentration is not changed, so there is no guanidinium gradient and the outward-facing proton gradient should drive guanidinium into the liposomes. The negative on-current indicates that charge is moving out of the liposome, consistent with the expected 2H^+^(out):1Gdm^+^(in) antiport (29). In Figure 1*B*, the activating buffer simultaneously sets an eightfold outward-facing guanidinium gradient in addition to the twofold outward-facing proton gradient. Under these conditions, the large guanidinium gradient drives uphill proton transport into the liposomes and reverses the direction of net charge movement. In the third and final stage, current flux in the opposite direction from the on-currents is observed when nonactivating buffer is reapplied to the sensor. While the off-currents in our experiments behave as expected qualitatively, we use only the on-currents for analysis following general practice in SSME data analysis (27).

Both chemical gradients and transmembrane voltage contribute to the total electrochemical potential driving transport. In a traditional reversal-potential assay, chemical gradients are kept constant, and transport is observed as a function of applied membrane voltage. In contrast, there is no applied membrane voltage in our assays, and instead, chemical gradients are varied (30). Thus, we plot the observed transport (integrated on-current) as a function of the applied chemical gradients, denoted as the ratio of the initial substrate and ion chemical potentials (Δ*μ*_*s*_/Δ*μ*_*i*_). Null transport at a given Δ*μ*_*s*_/Δ*μ*_*i*_ chemical potential ratio should correspond to an ion:substrate transport stoichiometry of the same value (see Experimental procedures for derivation). For Gdx’s 2H^+^:1Gdm^+^ transport stoichiometry, null transport should occur at a $\Delta \mu_{Gdm^{+}}/\Delta \mu_{H^{+}}$ ratio of 2. We chose chemical potential ratios corresponding to a variety of plausible H^+^:Gdm^+^ stoichiometries (Table 1) to determine the extent to which different stoichiometries can be distinguished by our method.

**Table 1:** Gdx reversal assay conditions

Figure	Internal conditions	External conditions					
Figure 1	pH 7.0	pH 7.3	pH 7.3	pH 7.3	pH 7.3	pH 7.3	pH 7.3
	1 mM Gdm^+^	1 mM Gdm^+^	500 μM Gdm^+^	353 μM Gdm^+^	250 μM Gdm^+^	125 μM Gdm^+^	62.5 μM Gdm^+^
$\Delta \mu_{Gdm^{+}}\left(\text{kJ}/\text{mol}\right)$		0	1.7	2.6	3.4	5.1	6.8
$\Delta \mu_{H^{+}}\left(\text{kJ}/\text{mol}\right)$		1.7	1.7	1.7	1.7	1.7	1.7
Figure 2, *B*, *C* and *E*	pH 6.7	pH 7.0	pH 7.0		pH 7.0	pH 7.0	
	1 mM Gdm^+^	1 mM Gdm^+^	500 μM Gdm^+^		250 μM Gdm^+^	125 μM Gdm^+^	
$\Delta \mu_{Gdm^{+}}\left(\text{kJ}/\text{mol}\right)$		0	1.7		3.4	5.1	
$\Delta \mu_{H^{+}}\left(\text{kJ}/\text{mol}\right)$		1.7	1.7		1.7	1.7	
Figure 2, *D* and *F*	pH 7.0	pH 7.35	pH 7.35		pH 7.35	pH 7.35	
	500 μM Gdm^+^	500 μM Gdm^+^	223 μM Gdm^+^		100 μM Gdm^+^	44 μM Gdm^+^	
$\Delta \mu_{Gdm^{+}}\left(\text{kJ}/\text{mol}\right)$		0	2		4	6	
$\Delta \mu_{H^{+}}\left(\text{kJ}/\text{mol}\right)$		2	2		2	2	
Potential ratio $\left(\Delta \mu_{Gdm^{+}}/\Delta \mu_{H^{+}}\right)$		0	1	3/2	2	3	4

### SSME can measure Gdx transport stoichiometry

Before proceeding to our analysis, we performed several critical controls. To assess the magnitude of signal due to solution exchange, we ran the assay using sensors prepared with two negative controls: proteoliposomes reconstituted with the nonfunctional mutant E13Q-Gdx and “empty” liposomes reconstituted without transporters. Signals for both negative controls were of similar magnitude and small compared with transport signals for WT-Gdx (Fig. 1*C* and Fig. S3). As an additional control, we prepared sensors from liposomes with different lipid-to-protein ratios (LPR) but with the same total amount of lipid. Altering the protein concentration in this manner will not alter the thermodynamics of transport but will affect transport kinetics as well as pre-steady-state currents related to electrogenic partial reactions, such as substrate binding or protein gating (27). Since we are interested in transport stoichiometry, it is important to check that the currents reflect the full transport cycle and not just a partial reaction. Integrated currents for WT-Gdx are independent of LPR (Fig. 1*D*), as expected if the currents correspond to transport.

Having confirmed that the currents reflect transport with the appropriate controls, we plotted transported charge as a function of chemical potential ratio. For both concentrations of WT-Gdx, null transport occurs at the expected 2:1 $\Delta \mu_{Gdm^{+}}/\Delta \mu_{H^{+}}$ ratio (Fig. 1*D*). This result is unchanged when either the empty liposome or E13Q controls are subtracted. This confirms that our assay accurately measures the transport stoichiometry of Gdx.

### Quantitative exchange of internal conditions

**Figure 2:**
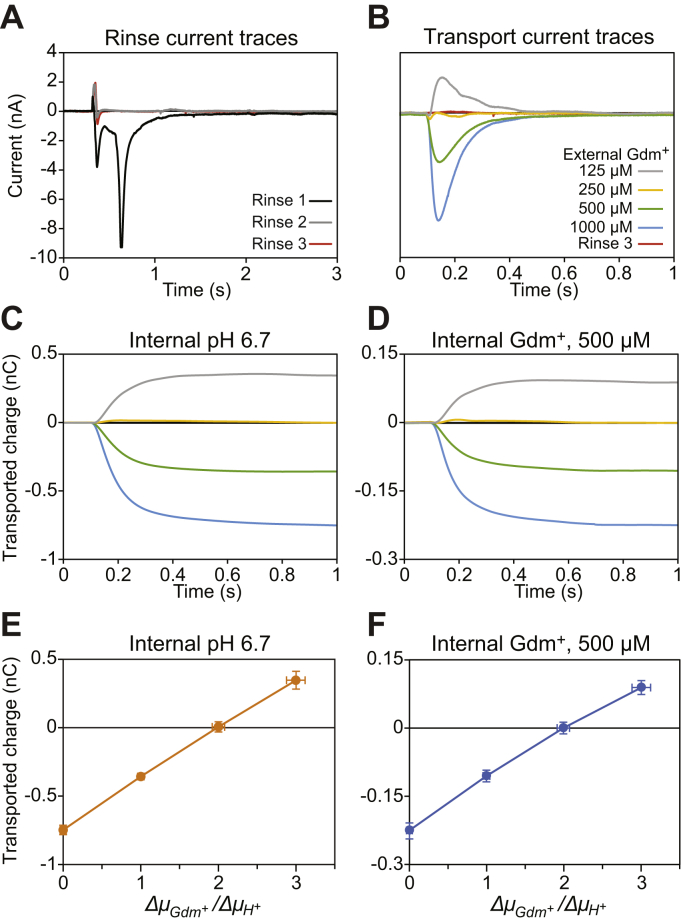
**Internal concentrations can be quantitatively changed on the sensor.***A*, plots of currents of successive rinses to change the internal buffer pH 7.00 and 1 mM guanidinium to pH 6.70 and 1 mM guanidinium. *B*, representative current traces for transport at the low pH condition compared with current from the final rinse. *C* and *D*, average integrated current traces for each internal buffer condition with signal from E13Q sensors subtracted. *E* and *F*, plots of transported charge *versus* potential ratios for an internal buffer conditions, with signal from E13Q sensors subtracted. Complete buffer conditions can be found in Table 1. Data points represent average normalized values obtained from four sensors for each sample condition. Y-error bars represent propagated standard error of the mean, and X-error bars are calculated by propagation assuming a 2% error in substrate and ion concentrations.

One key advantage of SSME over traditional liposomal assays is that one single SSM sensor allows dozens of measurements, drastically reducing sample requirements. Furthermore, it is well established that a single sensor can be used to screen different activating (external) buffers over several experiments (27, 31). We wished to test whether it was possible to reliably change the *internal* buffer solution of the liposomes adsorbed to the sensors, which would greatly expand the number of conditions that could be tested on a single sensor and further increase the throughput of this method. We prepared sensors at pH 7.00 with 1 mM guanidinium in the same manner as in the experiments in Figure 1. We then performed a series of rinses with pH 6.70, 1 mM guanidinium buffer while monitoring the current. After about 3 ml of total rinse volume, the currents stabilized near zero (Fig. 2*A*). We then proceeded to perform the assay with a nominal internal pH of 6.70 and activating buffers at pH 7.00. The integrated current was plotted against chemical potential, assuming that the transmembrane pH gradient matched the nominal value. The graph once again yielded a transport stoichiometry of 2H^+^:Gdm^+^ (Fig. 2, *C* and *E*), confirming that the internal pH was 6.70, as intended. This demonstrates that a single sensor can be used to test multiple internal pH conditions.

We then tested whether it is also possible to change the internal substrate concentrations. After preparing sensors with pH 7, 1 mM guanidinium buffer, we performed a series of three 1 ml rinses with pH 7.00, 500 μM guanidinium buffer, each separated by about 10 min, while monitoring the current. Several sensors required a fourth 1 ml rinse, but eventually, currents on all sensors stabilized near zero. Once the rinse currents stabilized at zero, we proceeded with the assay. The observed signal was lower overall, possibly the result of using lipids stocks that were 18 months old by the time of the experiment. Nevertheless, the signal trends were consistent with previous experiments, and once again, null transport occurred at the gradient ratio corresponding to 2H^+^:Gdm^+^ antiport (Fig. 2, *D* and *F*). This confirms that the internal Gdm^+^ was quantitatively exchanged on the sensor. These results provide evidence that internal substrate concentrations can be quantitatively exchanged on an SSME sensor, greatly expanding the possible range of experiments that can be performed using a single reconstitution of proteoliposomes.

### CLC-ec1 stoichiometry can be determined despite background signal

To determine whether the results of our assay with Gdx are generally applicable to other transporters, we next turned to CLC-ec1. This well-characterized transporter has previously been studied by SSME and is known to produce robust transport currents (32, 33). CLC-ec1’s canonical function is tightly coupled 1H^+^:2Cl^−^ antiport, but interestingly, it is also capable of transporting other anions with varying degrees of proton coupling (21). To test whether our SSME assay can accurately probe the effect of varied experimental conditions on transport stoichiometry, we performed a series of four reversal experiments with CLC-ec1 sensors (Table 2). Sensors with liposomes prepared from an identical reconstitution process without protein were used as negative controls.

**Table 2:** CLC-ec1 reversal assay conditions

Figure	Internal conditions	External conditions			
Figure 3 (blue)	pH 4.84	pH 5.24	pH 5.24	pH 5.24	pH 5.24
	150 mM chloride	150 mM chloride	111 mM chloride	95 mM chloride	60 mM chloride
$\Delta \mu_{Cl^{-}}\left(\text{kJ}/\text{mol}\right)$		0	0.74	1.12	2.25
$\Delta \mu_{H^{+}}\left(\text{kJ}/\text{mol}\right)$		2.26	2.26	2.26	2.26
Figure 3 (red)	pH 4.88	pH 4.21	pH 4.21	pH 4.21	pH 4.21
	3 mM chloride	3 mM chloride	5 mM chloride	6.5 mM chloride	14 mM chloride
$\Delta \mu_{Cl^{-}}\left(\text{kJ}/\text{mol}\right)$		0	−1.25	−1.89	−3.78
$\Delta \mu_{H^{+}}\left(\text{kJ}/\text{mol}\right)$		−3.79	−3.79	−3.79	−3.79
Figure 4 (orange)	pH 4.84	pH 4.17	pH 4.17	pH 4.17	pH 4.17
	15 mM nitrate	15 mM nitrate	25 mM nitrate	32 mM nitrate	70 mM nitrate
$\Delta \mu_{NO_{3}^{-}}\left(\text{kJ}/\text{mol}\right)$		0	−1.25	−1.86	−3.78
$\Delta \mu_{H^{+}}\left(\text{kJ}/\text{mol}\right)$		−3.79	−3.79	−3.79	−3.79
Figure 4 (purple)	pH 4.84	pH 5.23	pH 5.23	pH 5.23	pH 5.23
	15 mM nitrate	15 mM nitrate	11.2 mM nitrate	9.6 mM nitrate	6.1 mM nitrate
$\Delta \mu_{NO_{3}^{-}}\left(\text{kJ}/\text{mol}\right)$		0	0.72	1.10	2.21
$\Delta \mu_{H^{+}}\left(\text{kJ}/\text{mol}\right)$		2.20	2.20	2.20	2.20
Potential ratio $\left(\Delta \mu_{anion}/\Delta \mu_{H^{+}}\right)$		0	1/3	1/2	1

**Figure 3:**
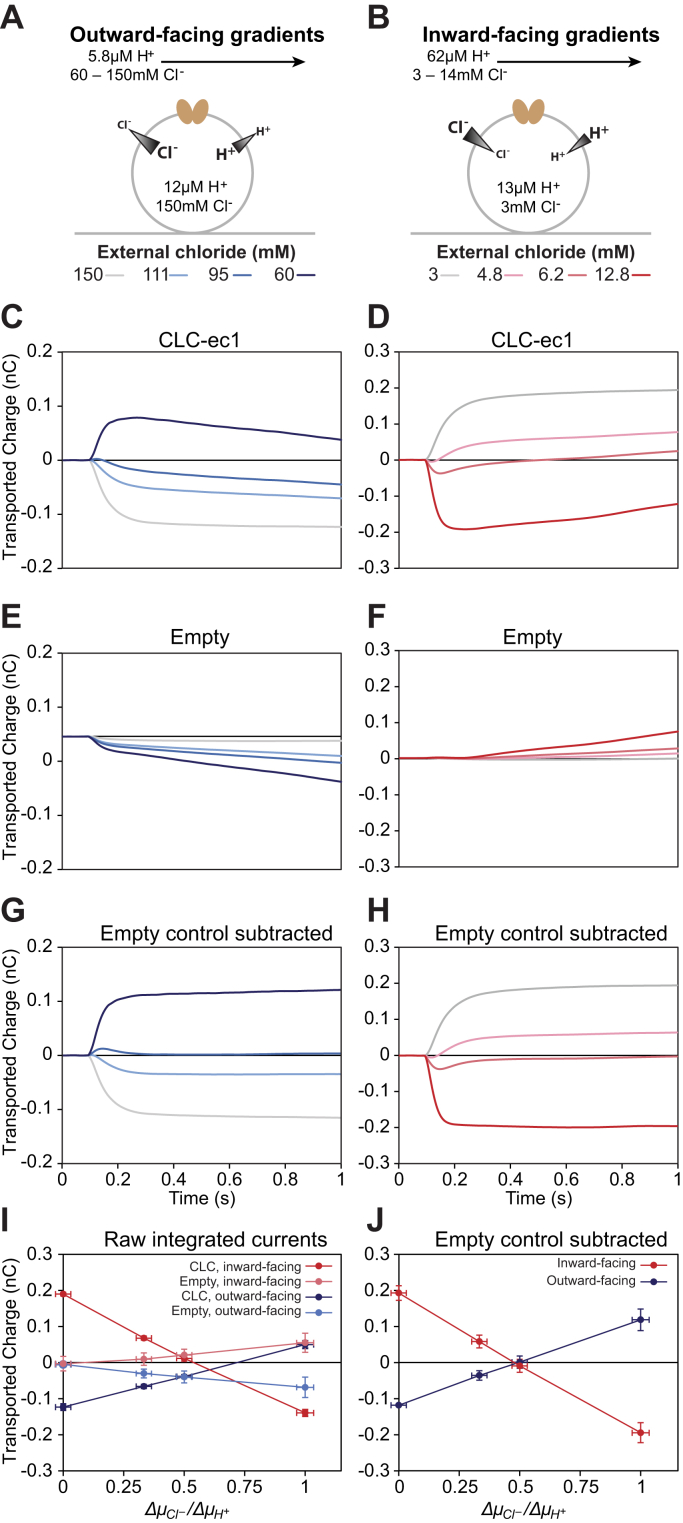
**CLC-ec1 chloride reversal assay.** The SSME reversal assay was performed with CLC-ec1 sensors with both outward-facing and inward-facing proton and chloride gradients. *A* and *B*, depiction of the internal and external buffer conditions during the on-current stage of the SSME assay. *C* and *D*, average integrated current traces for CLC-ec1 sensors. *E* and *F*, average integrated current traces for sensors prepared with empty liposomes. *G* and *H*, average integrated current traces with signal from empty liposomes subtracted. *I*, plots of transported charge *versus* chemical potential ratios for CLC-ec1 and empty liposomes with either outward-facing or inward-facing gradients. Empty liposome negative controls measured significant signal as the chloride gradient increased. *J*, correct stoichiometry of 1H^+^/2Cl^−^ is obtained after subtracting the negative control signal from the sample signal. For *I* and *J*, data points represent average normalized values obtained from four sensors for each (proteo)liposome sample. Y-error bars represent standard error of the mean, propagated where necessary, and X-error bars are calculated by propagation assuming a 1% error in chloride concentrations and 2% error in proton concentrations. Complete buffer conditions can be found in Table 2.

In the first set of experiments, the internal buffer matched the conditions of the reconstitution—pH 4.84 and 150 mM chloride. The external buffer was set to pH 5.24 and the external chloride concentration was varied to cover a range of possible proton:chloride stoichiometries (Fig. 3*A* and Table 2, row 1). Despite CLC-ec1’s high turnover rate, the observed signal did not reach a clear steady state under several experimental conditions. Instead, after an initial fast transport process, a slow but steady decrease could be observed in the integrated current traces (Fig. 3*C*). This decrease became more significant with larger chloride gradients, a phenomenon that was also observed with the empty liposome negative controls (Fig. 3*E*). This indicates that unlike with Gdx, a non-transporter-mediated process has a significant contribution to the signal, which must be taken into account when analyzing the data. Fortunately, the empty liposome control allows this signal to be isolated. Once isolated, background signals can be subtracted from sample signals to reveal the transporter-mediated signals in reversal potential assays (18) (see Supporting information for further discussion). When the signal in the empty liposome samples is subtracted from the signal in the CLC-ec1 samples, it is clear that CLC-ec1-mediated transport reaches steady state in fractions of a second (Fig. 3*G*), with null transport observed at the expected 1H^+^/2Cl^−^ antiport stoichiometry (Fig. 3, *G* and *J*).

**Figure 4:**
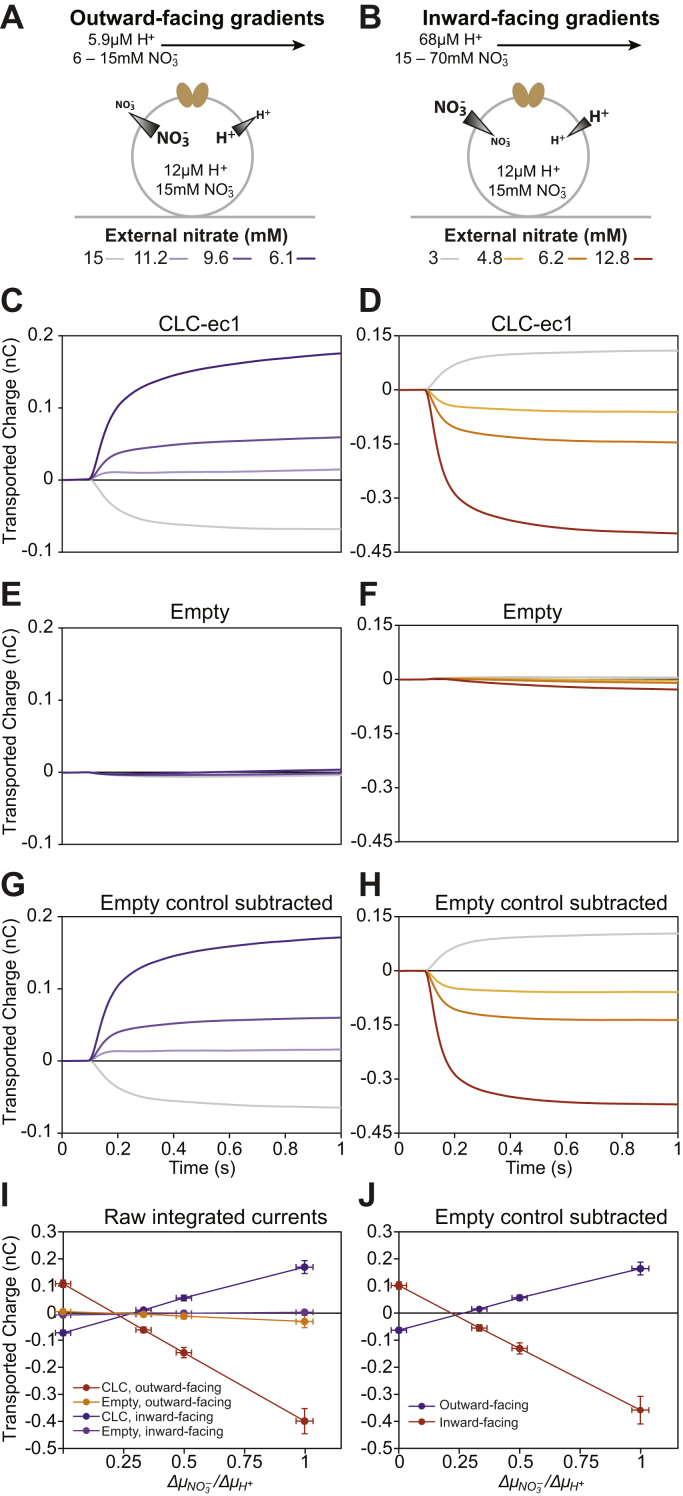
**CLC-ec1 nitrate reversal assay.** The SSME reversal assay was performed with CLC-ec1 sensors with both outward-facing and inward-facing proton and nitrate gradients. *A* and *B*, depiction of the internal and external buffer conditions during the on-current stage of the SSME assay. *C* and *D*, average integrated current traces for CLC-ec1 sensors. *E* and *F*, average integrated current traces for sensors prepared with empty liposomes. *G* and *H*, average integrated current traces with signal from empty liposomes subtracted. *I*, plots of transported charge *versus* chemical potential ratios for CLC-ec1 and empty liposomes with either inward-facing or outward-facing gradients. *J*, plots of integrated nitrate transport currents *versus* chemical potential ratios with the signal from empty liposomes subtracted. Reversal occurs at a potential ratio below 0.5, indicating that CLC-ec1 transports nitrate with decreased proton coupling compared with chloride, but the reversal point is too similar between the two conditions to distinguish between tightly coupled and loosely coupled transport. For *I* and *J*, data points represent average normalized values obtained from three sensors for each (proteo)liposome sample. Y-error bars represent standard error of the mean, propagated where necessary, and X-error bars are calculated by propagation assuming a 1% error in nitrate concentrations and 2% error in proton concentrations. Complete buffer conditions can be found in Table 2.

For the second set of experiments, the internal chloride concentration was reduced from 150 mM to 3 mM by successive washes of the desired internal buffer, as described above for Gdx (Fig. S5). We then proceeded with the second reversal experiment with inwardly directed proton and/or chloride gradients (Fig. 4*B* and Table 2, row 2). Once again, background signal was observed when chloride gradients were present (Fig. 3, *D* and *F*), but subtracting this signal from the signal of the CLC-ec1 sensors unambiguously yielded a 1H^+^:2Cl^−^ stoichiometry (Fig. 3, *H* and *J*). These results demonstrate the utility of our assay for a second system, even in the presence of significant background signal.

### Reduced proton:nitrate coupling by CLC-ec1 is due to nitrate leak

For the final two sets of reversal experiments, we rinsed the sensors to replace the chloride with 15 mM nitrate. CLC-ec1 transports nitrate with reduced proton coupling compared to chloride. Published reversal potentials are consistent with a transport stoichiometry between seven and ten nitrates per proton, but as Nguitragool and Miller noted, it is difficult to imagine a mechanism that could account for tightly coupled 7(+)NO_3_^−^:1H^+^ antiport (21). Subsequent computational and experimental studies on CLC-ec1 taken together support the suggestion that the increased nitrate:proton stoichiometry is due to presence of an uncoupled nitrate uniport pathway in parallel to the coupled nitrate:proton antiport pathway (33, 34, 35, 36). Nevertheless, tightly coupled transport has not been rigorously ruled out by published experimental data.

We performed nitrate reversal potential assays under two sets of conditions (Table 2)—first with inward-facing proton and nitrate gradients (Fig. 4, *A* and *C*–*J* orange) and second with outward-facing proton and nitrate gradients (Fig. 4, *B* and *D*–*J* purple). If our assay is capable of detecting the reduced nitrate:proton coupling, then the direction of transported charge should require a smaller anion gradient to reverse with nitrate than with chloride. Furthermore, if the reduced coupling is due to a combination of nitrate leak and coupled antiport and not tightly coupled nitrate:proton antiport with a large stoichiometry, we would expect the apparent stoichiometry to change as the experimental substrate and proton concentrations are altered (6, 21, 37) (see Supporting information and Fig. S6 for a more detailed explanation).

Our data shows that CLC-ec1 indeed transports nitrate with reduced proton coupling. When no nitrate gradient is present, the proton gradient dictates the direction of transported charge (Fig. 4 gray traces), but a relatively small nitrate gradient is sufficient for charge to be transported in the opposite direction of the proton gradient (Fig. 4 orange and purple traces) indicating a large apparent transport stoichiometry. However, our data is less clear on the question of whether this is due to tight coupling or nitrate leak. Any difference in the reversal point between the conditions is within the error of the measurement (Fig. 4, *I* and *J*). While the magnitude and direction of the gradients varied between the two conditions, both conditions had identical internal proton and nitrate concentrations. It is possible that these conditions were too similar to each other to distinguish the relative transport rates, so we adapted our assay to quickly survey a wider range of experimental conditions.

**Figure 5:**
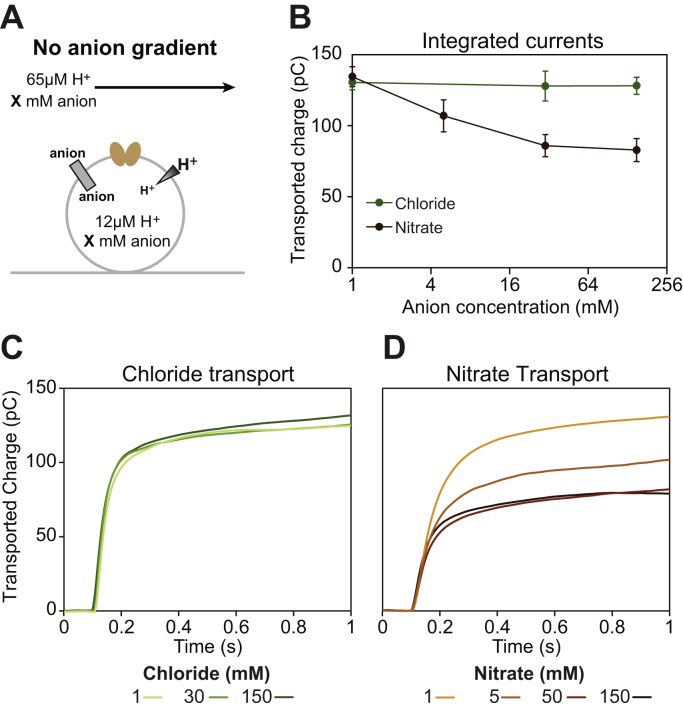
**CLC-ec1 transports nitrate with reduced proton coupling due to nitrate leak.** Sensors were successively equilibrated at seven different anion concentrations: 150 mM, 30 mM and 1 mM chloride, and then 1 mM, 30 mM, 150 mM, and 5 mM nitrate. For each data point, the external buffer was set to create an inward-facing proton gradient, but no anion gradient (*A*). With these conditions, the chemical potentials of the transported substrates are constant. Thus, if transport is tightly coupled, the amount of observed transport should not change with changing anion concentrations. If substrate leak is possible, it will occur in the opposite direction of the electrochemical gradients created by proton:anion antiport, reducing the signal. *B*, integrated current does not change as a function of chloride concentration but decreases as nitrate concentration increases. This confirms that chloride transport is tightly coupled and indicates that nitrate’s reduced proton coupling is due to nitrate leak. Data points represent average normalized values obtained from three sensors, with signal from empty sensors subtracted. Error bars indicate the standard error of the mean. *C*, average integrated current traces for chloride transport, with signal from empty liposome controls subtracted. *D*, average integrated current traces for nitrate transport, with signal from empty liposome controls subtracted.

We performed a series of measurements with a constant inward-facing proton gradient $\left(\Delta \mu_{H^{+}}\right)$ and matched internal and external chloride or nitrate concentrations (setting Δ*μ*_*anion*_ to zero) while varying the absolute value of the nitrate or chloride concentration (Fig. 5). If transport is tightly coupled, the amount of transport should be independent of the absolute anion concentration. On the other hand, anion leak should increase with increasing anion concentration (37). If anion leak is possible, anions will leak in the opposite direction of the electrochemical gradients created by proton:anion antiport, resulting in a decrease in observed transport. The observed signal was independent of chloride concentration between 1 and 150 mM, consistent with tightly coupled antiport of 1H^+^:2Cl^−^ by CLC-ec1. However, very different behavior was observed for nitrate transport. The observed signal decreased as the nitrate concentration increased, consistent with an increased prevalence of anion leak at higher nitrate concentrations. This provides clear evidence that the CLC-ec1’s reduced proton coupling with nitrate is due to nitrate leak rather than a higher order nitrate:proton stoichiometry.

## Discussion

As a key determinant of transporter function, accurate determination of transport stoichiometry provides great insight into transporter mechanism. Currently available methods for measuring transport stoichiometry are time-consuming and technically difficult, and as a consequence, transport stoichiometry is rarely quantitatively measured during structural or functional characterization of transporters. Among transporters that have had their transport stoichiometry characterized, many have been tested at a single experimental condition, which can lead to an assumption of a single transport stoichiometry that may not always be true (13, 21). Our SSME assay addresses several key technical obstacles for stoichiometry determination and in doing so, will facilitate measurement of transport stoichiometry for more transporters under a broader array of experimental conditions.

In addition to generalized signal detection and improvements to throughput, SSME also allows for fast and easy confirmation of the internal contents of the liposome. All that is required is to rinse the sensor with the desired internal buffer while recording. If the internal ion and substrate concentrations differ from the intended concentrations, a transport current will be evident (Fig. 2*A* and Fig. S5, blue traces), but no current will occur if the internal concentrations are matched to the known external buffer (Fig. 2, red traces and Fig. S5, yellow traces). This property can further be exploited to change the internal concentrations of the liposomes and test a wider variety of experimental conditions on the same sample, as demonstrated here for proton (Fig. 2), guanidinium (Fig. 2), chloride (Figs. 3 and 5), and nitrate (Figs. 4 and 5). It should be noted that both proton and chloride are relatively membrane-permeable (38, 39). Nevertheless, while the timescale of the internal buffer equilibration may increase for other molecules, recording the currents of successive rinses of internal buffer provides a robust method for ensuring that the internal buffer has indeed been exchanged.

The linear dependence of transported charge on ion/substrate potential hints at another advantage of our assay. In SSME, transport proceeds until sufficient membrane voltage is produced to oppose further transport—in effect, the reversal potential. While this reversal potential is not quantified directly by SSME, it is directly proportional to the total amount of transported charge. The linearity of the graphs in Figure 1, Figure 2, Figure 3, Figure 4 further indicates that the reversal potential is directly set by the ion and substrate concentration gradients (see Supporting information for further discussion). This facilitates experiments such as the one in Figure 5 that rely on the accurate analysis of transport potentials under different experimental conditions. In principle, similar experiments could be performed by running traditional liposomal transport assays to their endpoint, but such experiments are often rendered impractical due to the time they require or the presence of ion or substrate leaks (18). In contrast, the transport currents observed by SSME approach steady state in a fraction of a second, allowing rapid and accurate analysis of transport potentials.

As with any method, the reliability of this assay requires proper controls and assay conditions. SSME is sensitive enough to detect charge displacement due to conformational changes of proteins in the membrane (40), ion-transporter-binding events (41, 42), and solution exchange artifacts (27). To control for nontransport currents, sensors can be prepared with different LPRs, changing the protein concentration but keeping the lipid concentration constant. Currents due to binding or conformational changes will change with protein concentration, but integrated transport currents will not, as they depend only on the chemical potentials set by buffer exchange. In our Gdx results, there was no significant difference between the integrated currents for sensors prepared from LPRs of 150 and 400. As CLC-ec1 has previously been demonstrated to produce large transport currents by SSME (32), we did not perform assays with different CLC-ec1 LPRs. Furthermore, since CLC-ec1 concentrations were approximately an order of magnitude lower than the Gdx concentrations in our assay and since binding currents are proportional to protein concentration, it is reasonable to conclude that the currents in our CLC-ec1 assays are due to transport. Negative controls should be used to estimate background current due to solution exchange artifacts. For Gdx, it is possible to abolish transport activity through a single mutation (E13Q), but transport-dead mutants are not available for every transporter. In such cases, “empty” liposomes, which have undergone a simulated reconstitution process without protein, may be used instead. The simulated reconstitution process is crucial, as the addition and subsequent removal of detergent can greatly affect the integrity of the lipid bilayer. Empty liposomes that have undergone such a process can serve as quantitatively accurate negative controls, even in the presence of significant background signal, as shown in our chloride transport data for CLC-ec1.

It is also important to consider the effects of protein orientation in the membrane when purified transporters are reconstituted into proteoliposomes. For reversal-potential assays, it is generally assumed that the transport reaction is reversible and thus, that protein orientation does not matter. However, this assumption does not always hold, as has recently been demonstrated with the proton-coupled metal symporter DraNramp (15, 43, 44). Transporter orientation was addressed in two ways in our assays. First, the antiparallel structure of Gdx obviates the problem entirely, as the open-in and open-out conformations of the transporter are superimposable (29, 45, 46). Second, the CLC-ec1 assays were performed with both inward-facing and outward-facing gradients. If the orientation of reconstituted CLC-ec1 has a significant effect on transport function, the measured stoichiometry should vary when the gradient directions are flipped.

While this assay holds many improvements over traditional reversal potential assays, there are limits to the types of transport that it can measure. The most basic requirement is that the transport process must be electrogenic, though this is also a requirement of traditional reversal-potential experiments, as otherwise, changing the membrane potential will not affect the thermodynamics of the transport process. A second limitation is that while SSM electrophysiology can detect much smaller currents than traditional electrophysiological methods, currents from transporters with turnover rates below 1 per second may still be inaccessible through SSME (27, 28). We attempted to perform the assay with VcINDY but were unable to detect transport currents by SSME, perhaps unsurprisingly, given the reported turnover for VcINDY of 0.3 per minute (47). Signal from low-turnover transporters can be increased by lowering the LPR (48), but there is a limit to how much the concentration of protein can be increased while maintaining the integrity of the membrane. For transporters with turnover rates as low as VcINDY, the sensitivity provided by radioactivity may be necessary to observe reversal (22).

As our appreciation for the complexity of transporter mechanisms grows (3, 15, 16, 17), it is increasingly important to be able to measure transport stoichiometry. The SSME assay reported here addresses several key limitations of traditional reversal potential assays for measuring the stoichiometry of transporters. By directly measuring transported charge, this assay’s detection method is broadly applicable to electrogenic transporters that can be functionally reconstituted into liposomes. By allowing repeated measurements of the same sample, this assay reduces sample requirements and vastly increases throughput. And finally, by enabling quantitative exchange of internal substrate concentrations, this assay facilitates investigations of transporter behavior under a variety of experimental conditions. Taken together, these improvements will help to make transporter stoichiometry determination more routine, providing powerful tools for in-depth characterization of transporter mechanism and function.

## Experimental procedures

### Sample preparation

WT- and E13Q-Gdx were expressed in *E. coli* from a pET15b vector and purified as previously described for the homolog EmrE (45). Briefly, cells were lysed after overnight induction, and the protein was solubilized in 40 mM decylmaltoside (DM). Solubilized protein was run over a Ni^2+^-affinity column and eluted in 400 mM imidazole. The N-terminal hexahistidine tag was removed by overnight thrombin cleavage, and cleaved protein was further purified using size-exclusion chromatography. To minimize solution exchange artifacts, the buffers used for size-exclusion chromatography, reconstitution, and electrophysiology steps had the same salt composition: 50 mM MES, 50 mM MOPS, 50 mM bicine, 100 mM NaCl, and 2 mM MgCl_2_. Buffer pH values were carefully adjusted using only NaOH to ensure that internal and external Cl^−^ concentrations were identical for all measurements.

WT-Gdx or E13Q-Gdx was reconstituted into POPC proteoliposomes at a lipid-to-protein mole ratio of either 150:1 or 400:1 Gdx monomer (300:1 or 800:1 per functional dimer) in a pH 7.0 buffer. As an additional negative control, POPC liposomes were put through a simulated reconstitution process without protein. Detergent was removed with Amberlite XAD-2. Reconstituted liposomes were aliquoted and flash frozen. Immediately prior to measurements, liposome samples were thawed, diluted twofold in pH 7.00 buffer containing 2 mM guanidinium, and briefly sonicated. Ten microliter of liposomes at a lipid concentration of 1.4 μg/μl was then used to prepare sensors for each sample condition.

CLC-ec1 was purified as previously described (33, 49) and reconstituted into *E. coli* polar lipids (Avanti) in a pH 4.8 buffer containing 100 mM sodium citrate, 150 mM sodium chloride, 150 mM sodium isethionate, and 5 mM magnesium sulfate at a lipid-to-protein weight ratio of 50:1 (around 3300:1 mol ratio, assuming an average lipid molecular weight of 750). As a negative control, *E. coli* polar lipids were put through a simulated reconstitution process without protein. Detergent was removed by dialysis with four changes of the pH 4.8 buffer containing 100 mM sodium citrate, 150 mM sodium chloride, 150 mM sodium isethionate, and 5 mM magnesium sulfate.

Three millimeter gold electrode sensors were prepared according to the standard previously described protocol (27). Briefly, sensors were incubated for at least 30 min in an octadecane thiol solution, then rinsed thoroughly with isopropanol and water. The SSM was prepared by pipetting 1.5 μl of diphytanoyl phosphatidylcholine dissolved in n-decane onto the electrode surface, followed by 60 μl of an aqueous buffer. Immediately prior to measurements, liposome samples were thawed and briefly sonicated. Ten microliter of liposome sample was pipetted onto each SSM sensor and samples were adsorbed by centrifugation at 2500*g* for 30 min.

### Transport buffer preparation

Transport buffers were prepared by mixing buffer stock solutions. For the Gdx transport assays, buffer stocks were prepared at pH 6.7, 7.0, and 7.3 with either 2 mM HCl or 2 mM guanidinium-HCl for a total of six buffer stocks. Each buffer stock contained 50 mM MES, 50 mM MOPS, 50 mM bicine, 100 mM NaCl, and 2 mM MgCl_2_ and was adjusted to the desired pH with sodium hydroxide. To prepare transport buffers for each guanidinium concentration, the 2 mM guanidinium stock buffer was mixed with the 2 mM HCl stock buffer at the desired pH to ensure a constant chloride concentration.

For the CLC-ec1 transport assays, a total of nine buffer stocks were prepared: 300 mM stocks of sodium chloride, sodium nitrate, or sodium isethionate at pH 4.2, 4.8, or 5.2. In addition to the 300 mM sodium salt, each stock contained 100 mM citric acid and 5 mM magnesium sulfate. Stocks of each of the salts were adjusted to the desired pH values with sodium hydroxide. To prepare transport buffers of the desired substrate concentration, the 300 mM sodium isethionate stock was mixed with either the 300 mM sodium chloride stock or the 300 mM sodium nitrate stock at the desired pH to ensure a constant sodium and total anion concentration. Buffer conditions for each data point can be found in Tables 1 and 2 for Gdx or CLC-ec1, respectively.

### SSME data acquisition and analysis

All electrophysiology measurements were recorded on a Surfe2r N1 solid-supported membrane-based electrophysiology instrument from Nanion Technologies GmbH. Prior to recording any transport measurements, sensors were rinsed with at least 1 ml of nonactivating (internal) buffer while recording currents to ensure a flat baseline. Transport recordings occurred in three 1-s stages according to Figure 1 with 200 μl/s buffer perfusion. After each transport measurement, sensors were again rinsed with 1 ml of nonactivating buffer to ensure equilibration of the internal buffer before the next measurement.

Initial data analysis was performed using the Surfe2r N1 instrument-specific analysis software from Nanion. The final 200 ms of nonactivating buffer perfusion was averaged to obtain the baseline. Both peak current and integrated current data were obtained solely from the activating buffer perfusion. For transport currents with both positive and negative components (*e.g.*, the 250 μM trace in Fig. S3, *A* and *B*), the peak with the largest absolute value was recorded as the peak current. The entire stage of activating perfusion was integrated to obtain the integrated current values.

At least three sensors were prepared for each sample. While total current varied between sensors, transport behavior was highly consistent between sensors (Figs. S1 and S2). Reported peak and integrated currents consist of the average of at least three sensors, normalized to the average total peak, or integrated current observed on each sensor. Total current was determined by summation of the absolute value of the peak or integrated current observed on a sensor across all conditions tested. Y-error bars represent the standard error of the mean. X-error bars were calculated by propagation from Equation 8, assuming either a 1% (for CLC-ec1 buffers) or 2% (for Gdx buffers) error in substrate concentration and a 2% error in proton concentration.

### Derivation of transport equations

We assume that transport proceeds according to a stoichiometric transport reaction:

(1) $$nIon_{out}+mSubstrate_{out}⇄nIon_{in}+mSubstrate_{in}$$

If *n* and *m* are the same sign, this equation describes symport while if *n* and *m* are opposite signs, the equation describes antiport. Note that if *n* and *m* are opposite signs, Equation 1 can be rearranged to give an equation for antiport with positive stoichiometric coefficients:

(2) $$\;nIon_{in}+mSubstrate_{out}⇄nIon_{out}+mSubstrate_{in}$$

The chemical potential of ion and substrate across the membrane is given by:

(3) $$\;\text{Δ}\mu_{i}=RT\text{ln}\left(\frac{{\left[Ion\right]}_{in}}{{\left[Ion\right]}_{out}}\right)+z_{Ion}F\text{ΔΨ}$$

(4) $$\;\text{Δ}\mu_{s}=RT\text{ln}\left(\frac{{\left[Substrate\right]}_{in}}{{\left[Substrate\right]}_{out}}\right)+z_{Substrate}F\text{ΔΨ}$$

At time zero in our SSME assay, there is no membrane voltage. Thus, the chemical potentials of the ion and substrates are described completely by their respective gradients:

(5) $$\;\text{Δ}\mu_{i}=RT\text{ln}\left(\frac{{\left[Ion\right]}_{in}}{{\left[Ion\right]}_{out}}\right)$$

(6) $$\;\text{Δ}\mu_{s}=RT\text{ln}\left(\frac{{\left[Substrate\right]}_{in}}{{\left[Substrate\right]}_{out}}\right)$$

The free energy for the coupled transport reaction is given by:

(7) $$\;\text{ΔG}=n\text{Δ}\mu_{i}+m\text{Δ}\mu_{s}$$

When ΔG=0,

(8) $$-\frac{n}{m}=\frac{\text{Δ}\mu_{s}}{\text{Δ}\mu_{i}}=\frac{RT\text{ln}\left(\frac{{\left[Substrate\right]}_{in}}{{\left[Substrate\right]}_{out}}\right)}{RT\text{ln}\left(\frac{{\left[Ion\right]}_{in}}{{\left[Ion\right]}_{out}}\right)}$$

Thus, plotting transported charge against Δ*μ*_*s*_/Δ*μ*_*i*_ gives an x-intercept at −*n*/*m*, allowing determination of the transport stoichiometry. For more complete mathematical descriptions of transport equations, see previous publications (18, 50, 51).

## Data availability

All data needed to evaluate conclusions of the manuscript are present in the paper and/or supplementary materials. Requests for raw data should be submitted to KHW.

## Supporting information

This article contains supporting information.

## Conflict of interest

The authors declare that they have no conflicts of interest with the contents of this article.
