# Supplementary material for: A solid-supported membrane electrophysiology assay for efficient characterization of ion-coupled transport
Source: J Biol Chem. 2021 Sep 23;297(4):101220. doi: 10.1016/j.jbc.2021.101220 (PMC8517846; doi:10.1016/j.jbc.2021.101220)
Supplement: Supporting information [file mmc1.docx]

**Supporting Information**

*A note on replicates and reproducibility*

As we collected our data, we observed several trends which impacted our subsequent data analysis. All Gdx data were collected using four sensors per sample condition (LPR150, LPR400, etc.). Each sensor was then used to record two traces per chemical potential ratio, which allowed us to assess the reproducibility of the data both from different recordings on the same sensor and from recordings of the same conditions across different sensors. Different traces from the same sensor overlay strikingly well (Fig. S1A), but there is variability in the amount of signal observed across different sensors (Fig. S1B). This is not surprising, as the amount of signal depends on the number of liposomes that are adsorbed to the electrode surface during sensor preparation. Nevertheless, the signal trends across sensors are consistent (Figs. S1C, S1E, and S2). Normalizing the signal observed on a given sensor to the average total signal from that sensor accounts for the variation between different sensors (Fig. S1 D,F), and thus, data normalization was performed for all samples.

*Analysis of integrated current vs. analysis of peak current*

We analyzed the results of changing the applied proton and substrate gradient on observed current using two methods: peak on-current (Fig. S3 and S4) and integrated on-current (Fig. 1). Peak on-current is a parameter related to the turnover rate of the transporter and is a commonly quantified for SSME experimental analysis (27, 28). However, peak current is ultimately a kinetic parameter, while transport stoichiometry is a thermodynamic quantity. We therefore considered a second parameter by analyzing the integrated current, representing the total net charge movement, as a function of the chemical potential ratio.

The peak current reverses near the expected potential, but changes with both protein concentration and the type of negative control (Fig. S4B). This renders it difficult to distinguish between the previously reported 2H^+^:1Gdm^+^ antiport stoichiometry and a less likely but still theoretically possible 3H^+^:2Gdm^+^ antiport stoichiometry. However, plotting the integrated on-current as a function of chemical potential ratio clearly and precisely yields the published 2H^+^:1Gdm^+^ transport stoichiometry (Fig. 1D). This result is unchanged when either the empty liposome or E13Q controls are subtracted. This confirms that the thermodynamic parameter of integrated current is the better metric for determining transport stoichiometry in this assay.

*Effect of experimental conditions on transport stoichiometry*

Untangling transporter-mediated leak from coupled transport is a different proposition than separating coupled transport from a background signal, as transporter-mediated leak cannot be isolated with negative controls. Instead, it is helpful to consider the relationship between the kinetic mechanism and the transport thermodynamics of a loosely coupled system. Figure S6 shows simple kinetic models for both tightly coupled (Fig. S6A) and loosely coupled (Fig. S6B) transporters. In a tightly coupled system, there is only one transport pathway, setting a single transport stoichiometry that is invariant across experimental conditions. In contrast, the loosely coupled model allows both coupled antiport (red cycle) and leak (blue cycles). The apparent transport stoichiometry in such a system is a function of the relative flux through the coupled and uncoupled (leak) pathways, which depends on the relative rates of substrate binding, substrate release, and alternating access. Changing the proton or substrate concentrations will change the substrate binding rates, altering the partitioning between pathways, the net flux through leak and transport cycles, and the apparent net stoichiometry (6, 21, 37).

*Goldman-Hodgkin-Katz reversal potential and subtracting background signal*

The ability to quantitatively exchange the internal liposomal contents indicates that the integrity of the lipid bilayers in our samples are imperfect. While this property is not unique to SSME (no lipid bilayer is completely impermeable to ions), it is important to consider the implications that ion leak currents have on our assays. In SSME, charge transport proceeds until the membrane voltage reaches the reversal potential (32). The Goldman-Hodgkin-Katz (GHK) voltage equation (Eq. S1) describes the reversal potential in terms of the relative flux of permeant ions (50). For monovalent ions, the equation can be written:

(S1) $E_{rev}=\frac{RT}{F}ln\left( \frac{\sum_{i}^{n} P_{C_{i}^{+}}\left( C_{i}^{+} \right)_{out}+\sum_{j}^{m} P_{A_{j}^{-}}\left( A_{j}^{-} \right)_{in}}{\sum_{i}^{n} P_{C_{i}^{+}}\left( C_{i}^{+} \right)_{in}+\sum_{j}^{m} P_{A_{j}^{-}}\left( A_{j}^{-} \right)_{out}} \right)$

Where E_rev_ is the reversal potential, R is the universal gas constant, F is Faraday’s constant, C and A are monovalent cations and anions, respectively, and P is the relative permeability of each ion. This equation holds true as long as the concentrations of the permeable ions are constant. While transport must change ion concentrations to some extent, the relative linearity of the graphs of transported charge against substrate potential (Fig. 1D, 2E, 2F, 3J, and 4J) indicates that the equilibrium voltage is a direct, linear function of the initial chemical gradients. Thus, the concentrations must not change significantly during the one second that transport is measured, and the GHK equation can be applied.

The relevant takeaway from the GHK equation for our assay is that ion permeability through the lipid bilayer should have a predictable impact on the amount of observed transport. If bilayer permeability is much slower than permeability through the transporter, the observed transport at a given condition will be a function only of the stoichiometry of the transporter. This is what we observe with Gdx, where currents in the negative controls are minimal compared to currents in samples containing active transporter. At the other extreme, if bilayer permeability is fast relative to coupled transport, then leak currents will dominate and transport currents will be unobservable. This explains why stoichiometry determination using our SSME assay fails for low turnover transporters such as VcINDY. In between these two extremes are examples such as CLC-ec1, where transport is faster than leak, but leak currents are still observable on the timescale of the measurements. The GHK equation indicates that signal from the coupled transport process can still be determined under these conditions, as long as the signal due to bilayer permeability can be isolated and subtracted. This is precisely what we observe with CLC-ec1. Subtracting the empty liposome signal from the CLC-ec1 signal unambiguously yields CLC-ec1’s 2Cl^-^:1H^+^ antiport stoichiometry.

Additional experimental conditions can help determine when leak currents can be subtracted versus when they are too large to be dealt with effectively. Changing permeant ion concentrations while maintaining the ion gradient ratios will change the relative rates of transport and leak currents and thus, change the GHK reversal potential. While the thermodynamics of the system will be altered, the thermodynamics of a tightly coupled transport process will be constant. Once again, this is borne out by the CLC-ec1 data. When only the CLC-ec1 liposome signals are considered, the null transport point varies significantly between the outward-facing (150 mM internal chloride, Fig. 3I, dark blue) and inward-facing (3 mM internal chloride, Fig. 3I, dark red) gradient conditions. However, the empty liposome controls allow the background signal to be isolated, and the difference between conditions disappears when the empty liposome signals are subtracted (Fig. 3J).


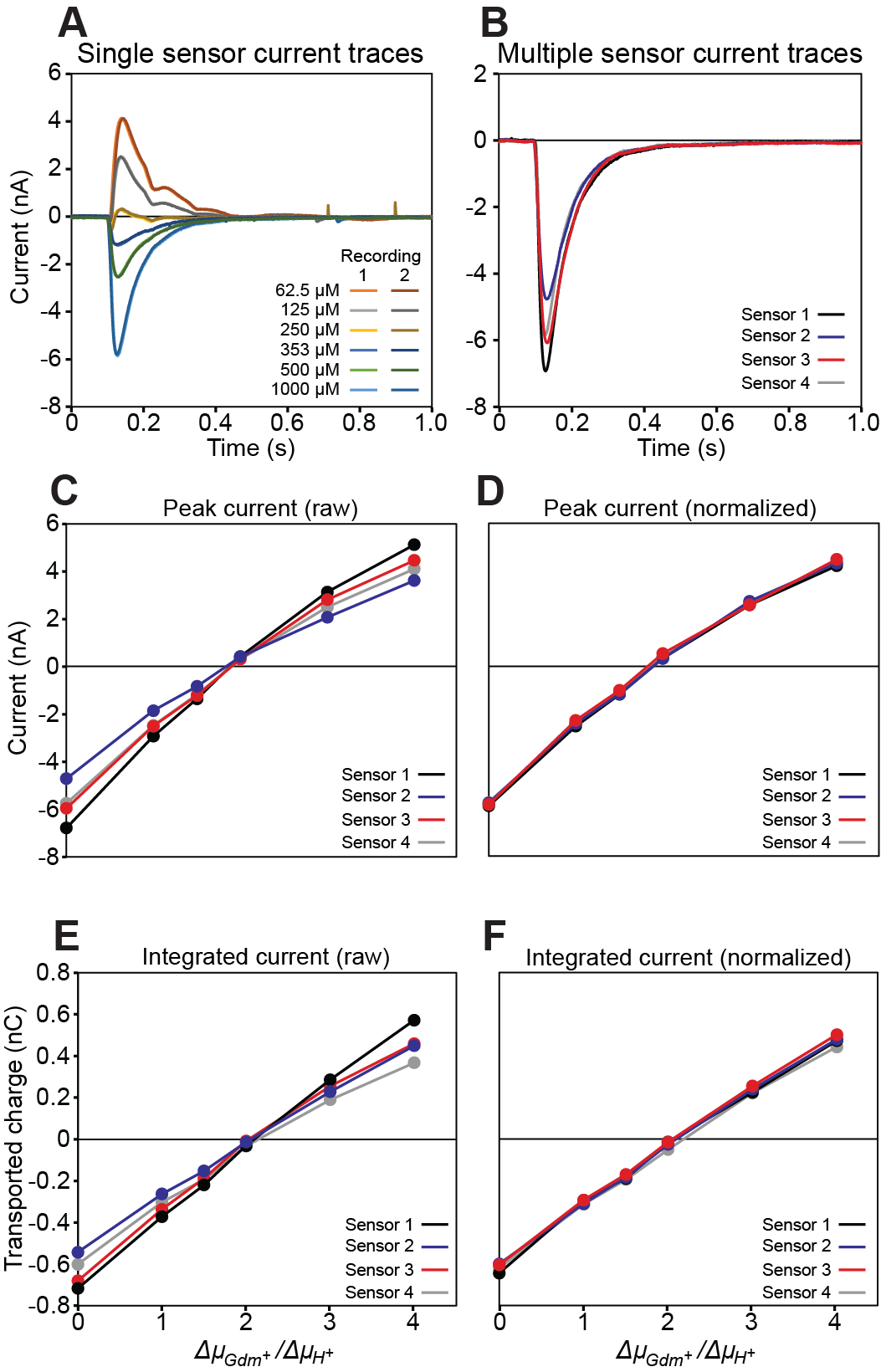


**Fig. S1** **– Examples of replicates and data normalization.** **(A)** Representative current traces showing two recordings of a single sensor for each external buffer condition. Current traces for the same condition overlay nearly perfectly, indicating that little variability is introduced from repeated recordings on a single sensor. **(B)** Current traces for a single condition (1 mM Gdm+, pH 7.3 external) for four different sensors indicate that there is variability in signal intensity across different sensors. Nevertheless, each sensor exhibits the same trends in both peak current **(C)** and integrated current **(D)** across the different experimental conditions. Normalizing the total signal observed on each sensor to the average total signal observed across sensors accounts for most of the variability observed between sensors in both peak **(E)** and integrated **(F)** currents. All data shown is for WT-Gdx LPR 400 liposomes. The same trends in data reproducibility were observed for all sample conditions.


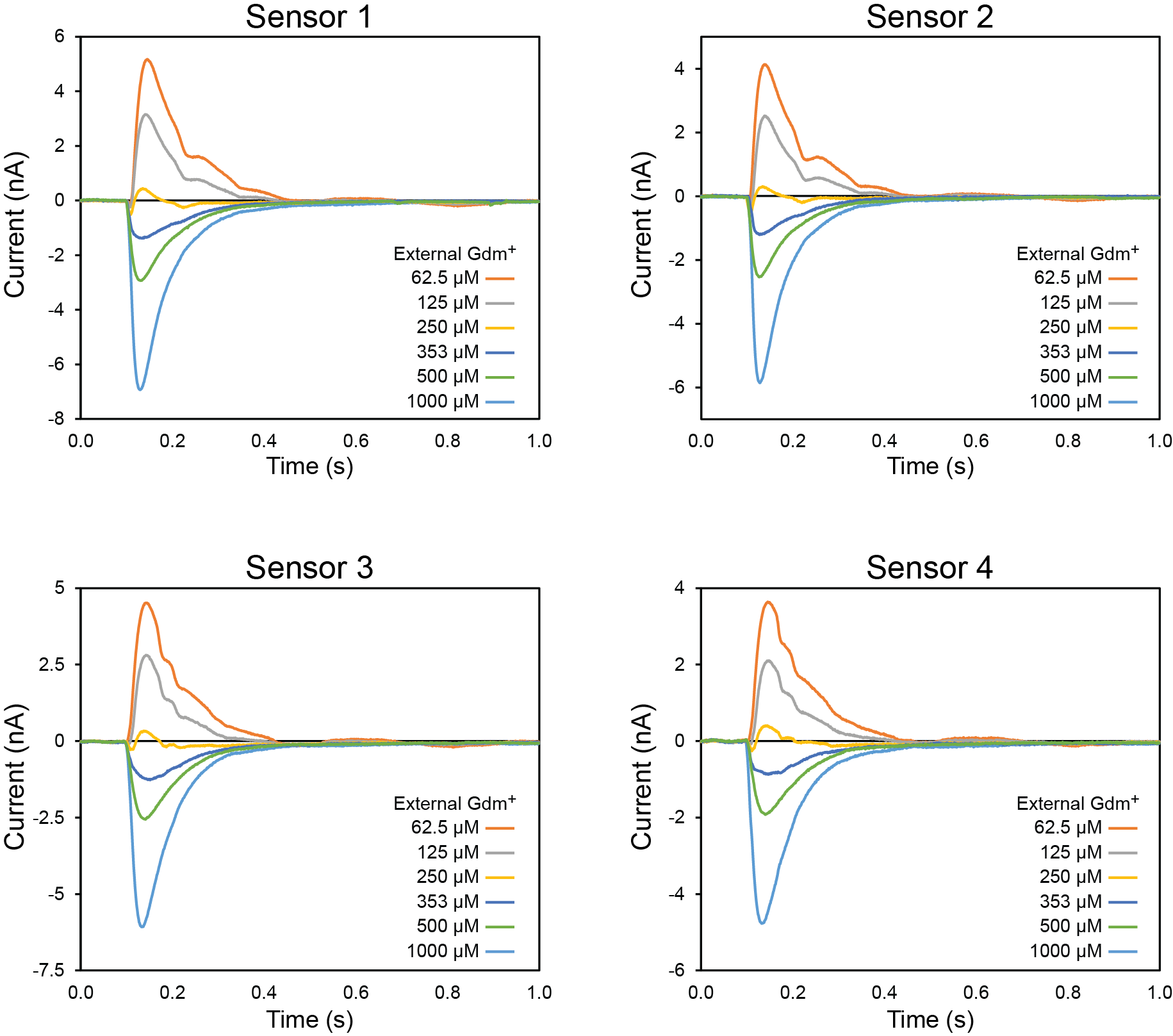


**Fig. S2** – *Current traces from all WT Gdx LPR 400 sensors*. Amount of transport varied across sensors(note the variation in the scale of the y-axis), but signal trends across different external buffer conditions is consistent from sensor to sensor.


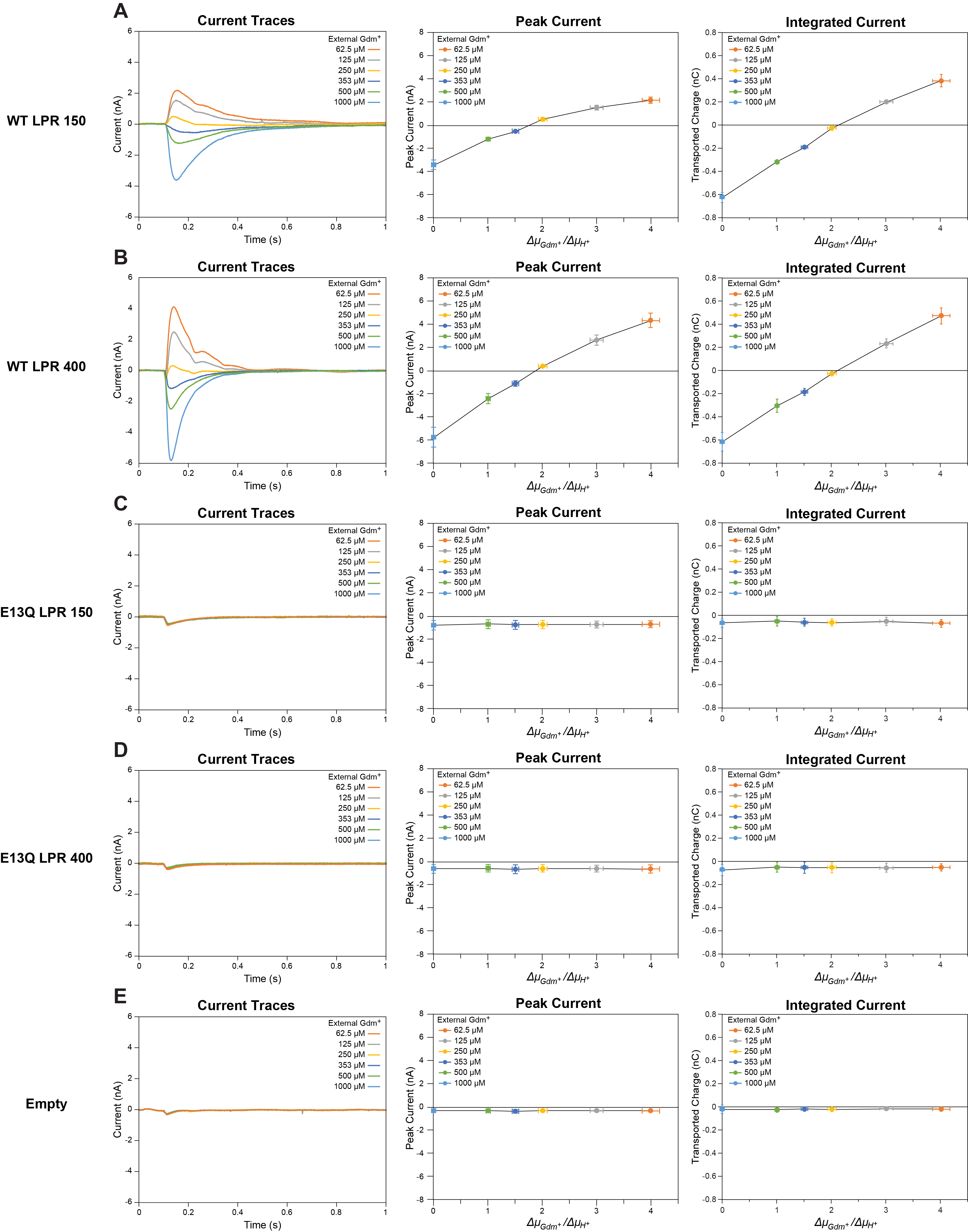


**Fig. S3** **– Reversal assay data across sample conditions.** Representative current traces (*left column*), average peak current (*middle column*), and average integrated current (*right column*) for each external guanidinium concentration are shown for WT-Gdx proteoliposomes with an LPR of 150 **(A)** and an LPR of 400 **(B)**, E13Q-Gdx proteoliposomes with an LPR of 150 **(C)** and an LPR of 400 **(D)**, and empty liposomes **(E)**. Data points represent average values obtained from four sensors for each (proteo)liposome sample. Y-error bars represent standard error of the mean and X-error bars are calculated by propagation assuming a 2% error in substrate and ion concentrations. Complete buffer conditions can be found in Table 1.


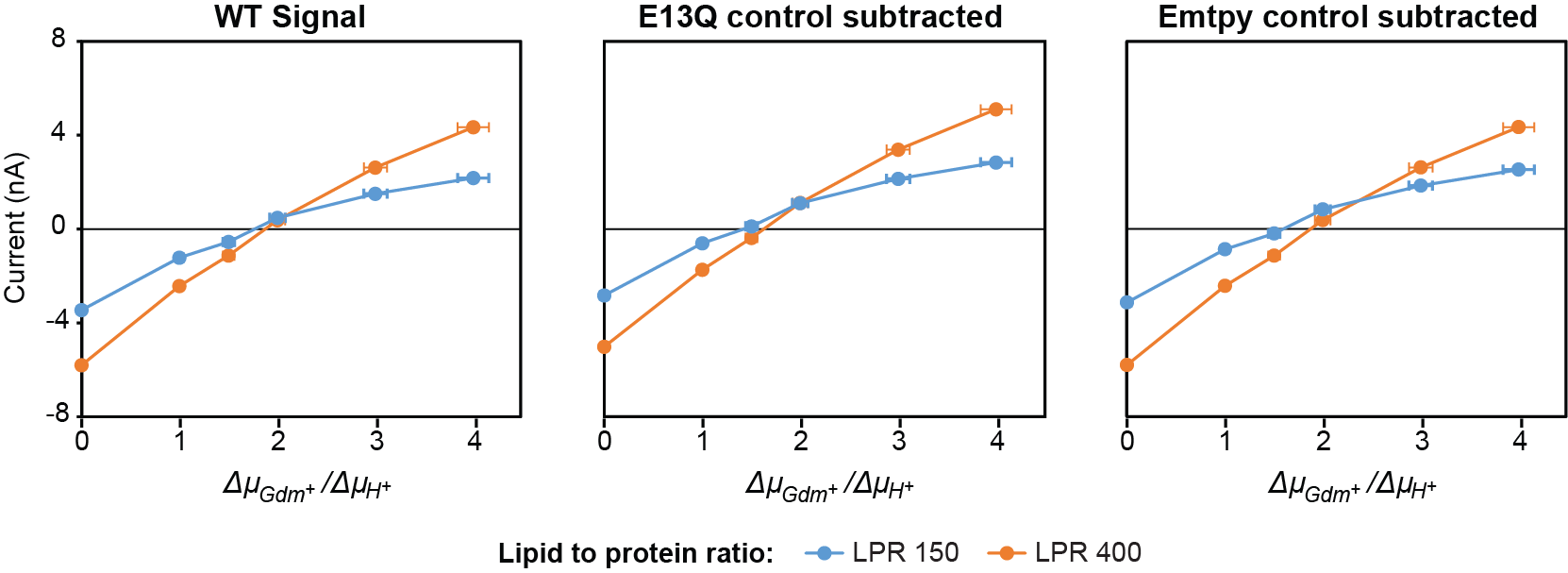


**Fig. S4** **– Peak current analysis yields the correct stoichiometry***.* Plots of peak currents against the imposed chemical potential ratio with the indicated data treatment. Peak current analysis yields a null current at a stoichiometry between 1.5 and 2H^+^/Gdm^+^, depending on the LPR and on which control (if any) is subtracted. Data points represent average normalized values obtained from four sensors for each (proteo)liposome sample. Y-error bars represent standard error of the mean, propagated where necessary, and X-error bars are calculated by propagation assuming a 2% error in substrate and ion concentrations. Complete buffer conditions can be found in Table 1.


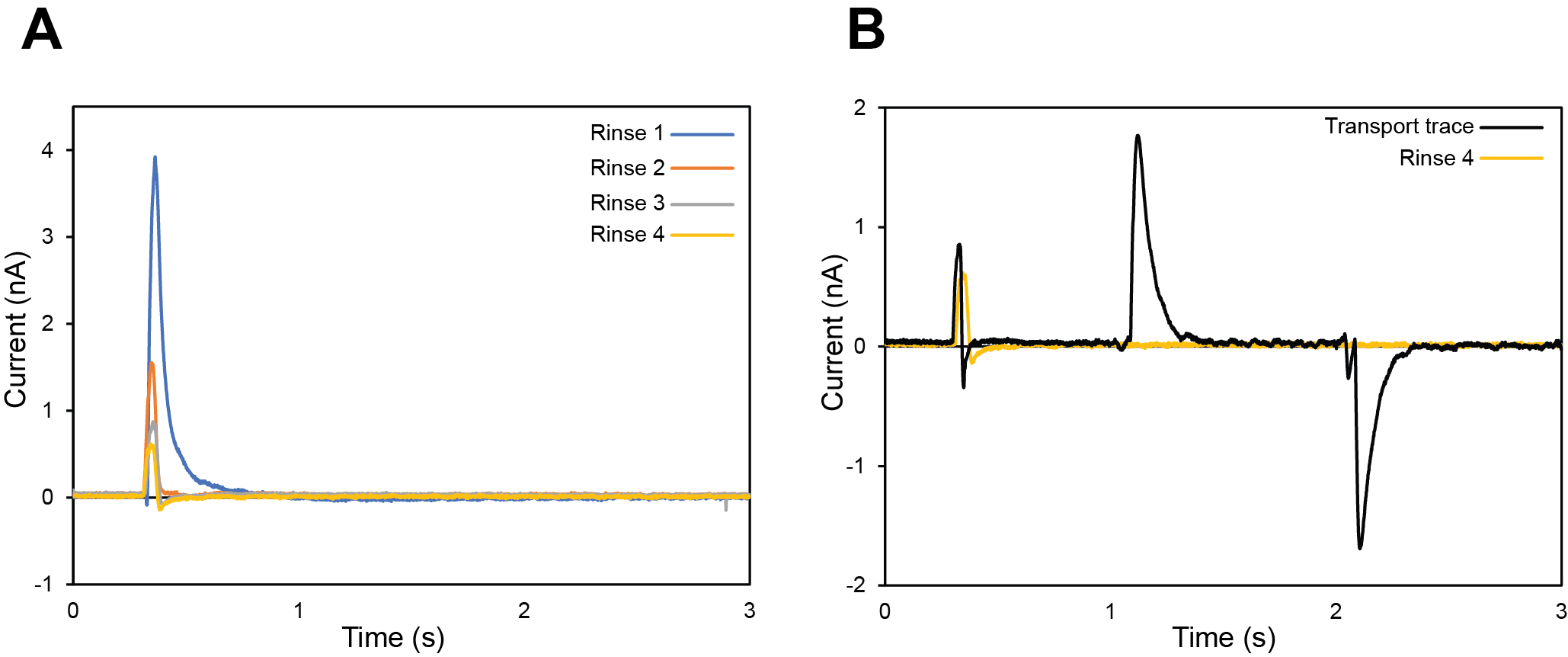


**Fig. S5 – Representative current traces of internal buffer exchange.** **(A)** Observed current reaches a baseline level after a sensor previously equilibrated with 150 mM chloride buffer undergoes four successive 600 μL rinses of 30 mM chloride buffer. **(B)** Overlay of the final buffer rinse and the subsequent transport current trace.


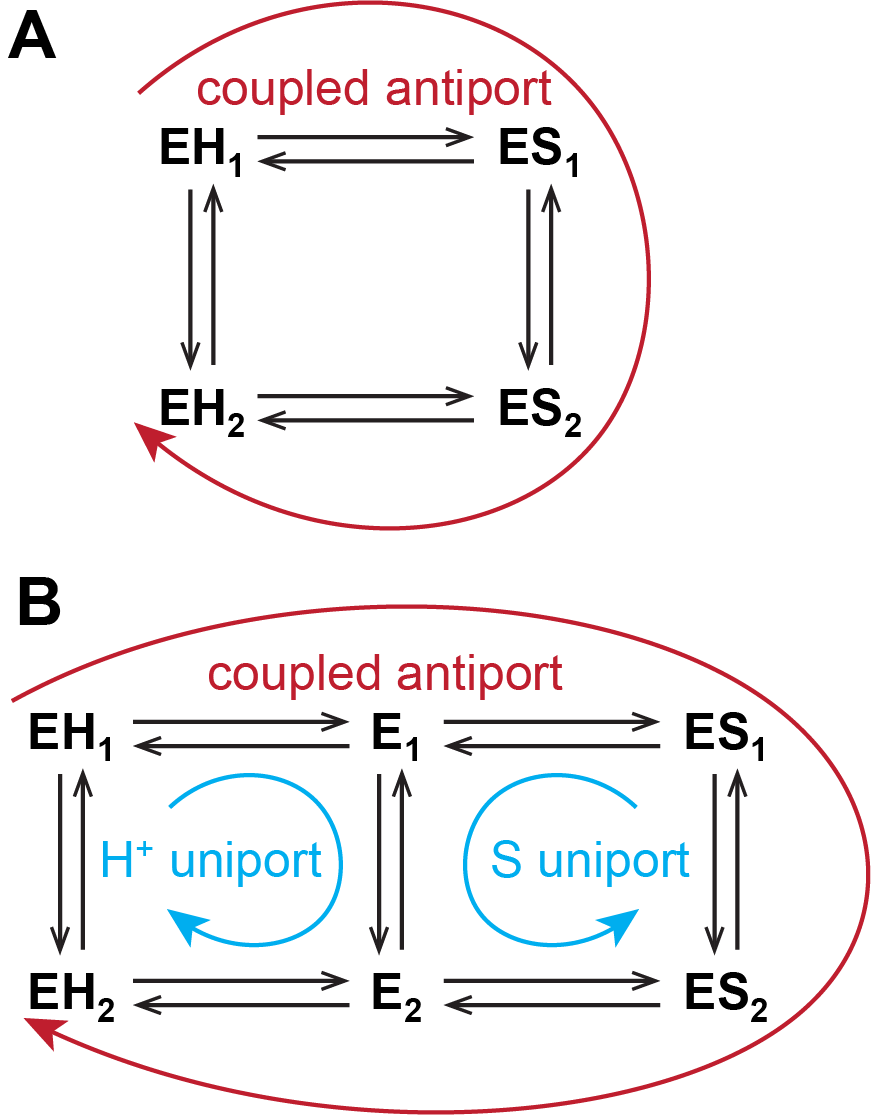


**Fig. S6** **– Transport models.** Depictions of simple kinetic models for tightly coupled **(A)** or loosely coupled **(B)** transport, where EH is proton-bound transport, ES is substrate-bound transporter, E is empty transporter, and the subscript indicates the orientation of the transporter with respect to the membrane. In **(A)**, only one transport cycle is available, setting a single transport stoichiometry. In **(B)**, coupled antiport is still possible (red cycle), but alternating access of the empty transporter can short-circuit transport, leading to proton and substrate leak cycles (blue).
